# The Interactions between Cells and Viruses

**DOI:** 10.3390/ijms25136886

**Published:** 2024-06-23

**Authors:** Masahiro Fujimuro

**Affiliations:** Department of Cell Biology, Kyoto Pharmaceutical University, Kyoto 607-8412, Japan; fuji2@mb.kyoto-phu.ac.jp

Many infectious diseases are caused by life-threatening DNA and RNA viruses and have been reported worldwide, including those caused by emerging and re-emerging viruses. Viral infections can cause a variety of diseases in their host species. Antiviral medications help to relieve the symptoms of some viruses by inhibiting viral enzymes or virus-mediated events. The past half century has seen the development of antiviral drugs and vaccines for several viral infections, including smallpox virus, human immunodeficiency virus-1 (HIV-1), hepatitis C virus (HCV), influenza, and some herpes viruses. However, there remain many serious and life-threatening viral infections for which no antiviral drugs have been clinically approved.

One of the key factors for a virus to achieve effective viral infection and viral replication is the interaction between cell and virus. This is achieved by dysregulation (or utilization) of cellular functions by viral proteins or viral microRNAs through their interactions with cellular molecules. Cellular functions that may be affected include signal transduction, gene expression, metabolic processes, post-translational protein modification, protein degradation, extra- or intra-cellular transport, organelle biogenesis, apoptosis control, and immune responses. Recent studies have revealed that viruses can hijack these cellular functions for the establishment of infection, the persistence of infection, prolonging their survival, controlling cell proliferation, preventing apoptosis, and evading the immune surveillance of infected cells. In this way, viruses manipulate various cellular functions to create a favorable environment for the virus. Conversely, host species exert antiviral effects during a viral infection through interactions between cell and virus. 

This article is intended to act as an introduction for this Special Issue. The Special Issue includes reports of recent findings relating to the molecular mechanisms necessary to establish effective viral infection and replication (or host immune responses) through interactions between cellular and viral molecules. 

Rabies is a potentially deadly viral disease caused by rabies virus (RABV). It is transmitted through the bite of an infected animal, resulting in irreversible neurological symptoms and a 100% fatality rate in humans if not treated [1,2]. In one of the manuscripts included in this Special Issue, a human-induced pluripotent stem cell (iPSC)-derived neuron model for the molecular study of RABV and host neuronal cell interactions was established and reported by Chailangkarn et al. [3]. They showed that RABV infection induced temporal changes in proteins related to metabolic processes, immune responses, the neurotransmitter transport/synaptic vesicle cycle, cytoskeleton organization, and cell stress responses. In addition, they reported that HSPA8, which belongs to the HSP70 family, is involved in RABV replication and pathogenesis.

Porcine circovirus 2 (PCV2) and pseudorabies virus (PRV) coinfection causes severe neurological and respiratory symptoms and high mortality in piglets [4,5]. However, the mechanism involved in PRV and PCV2 coinfection and its pathogenesis remains largely unknown. Li et al. reported that PRV and PCV2 coinfection promoted the activation of NF-κB, c-Jun N-terminal protein kinases (JNKs), p38 MAPK, and NOD-like receptor protein 3 (NLRP3) pathways, along with the expression of interferon (IFN)-γ, IFN-λ1, IFN-stimulated gene 15 (ISG15), interleukin (IL)-6, and IL1β [6]. Meanwhile, this coinfection downregulated the JAK/STAT pathway, the ERK pathway, and the expression of IFN-β and tumor necrosis factor (TNF)α. Thus, the coinfection of PRV and PCV2 enhances immunosuppression and inflammation through the dysregulation of these cellular signaling pathways. Chen et al. also reported that PRV and PCV2 coinfection induced endoplasmic reticulum (ER) stress in host cells and activated unfolded protein response (UPR), including the PERK-eIF2α-ATF4-CHOP pathway and IRE1-XBP1-EDEM pathway, resulting in cell apoptosis relating to exacerbating the disease [7].

The sodium taurocholate co-transporting peptide (NTCP) is an essential molecule for hepatitis B virus (HBV) entry into hepatic cells as well as binding with the HBV spike protein [8]. This finding led to the development of an in vitro HBV infection system using a human hepatocellular carcinoma (HCC) cell line expressing NTCP [9,10]; however, the key mechanism of HBV entry remains unknown. Ueda et al. identified ATP5B, a component of F1F0 ATP synthase, as another essential factor for HBV entry [11]. ATP5B is expressed on the cell surface of HCC cell lines. It can bind the myristoylated preS1 region of the HBV spike protein but not the non-myristoylated preS1 region. Moreover, the knockdown of ATP5B in NTCP-expressing HepG2 cells reduced HBV infectivity. These findings suggest that ATP5B is an essential factor for HBV entry into cells.

Kaposi’s sarcoma-associated herpesvirus (KSHV) is the causative agent of Kaposi’s sarcoma and primary effusion lymphoma [12]. During lytic infection, KSHV late genes are expressed by the virus-specific pre-initiation complex (vPIC), which consists of viral transcription factors (ORF34, ORF18, ORF30, etc.) and a cellular RNA polymerase II [13]. However, the nature of the protein–protein interaction of the vPIC factors has not been completely elucidated. Maeda et al. characterized the interaction of vPIC factors ORF18 and ORF30 using a bimolecular fluorescence complementation assay, a pull-down assay, and an AlphaFold2 predicted binding model [14]. As a result, they identified four amino acid residues (Leu29, Glu36, His41, and Trp170) of ORF18 that were responsible for its interaction with ORF30.

Epstein–Barr virus (EBV) is closely associated with Burkitt lymphoma, nasopharyngeal carcinoma, and gastric carcinoma [15]. EBV infection has been identified in approximately 9% of gastric cancers, and EBV-associated gastric carcinoma (EBVaGC) is a common cancer among EBV-related malignancies [16]. As DNA methylation of both viral and host DNA is one of the major mechanisms involved in the development of EBVaGC [17], demethylating agents may be utilized as novel therapeutic agents for EBVaGC. The effects of the demethylating agent MC180295 on cell growth of the EBVaGC cell lines YCCEL1 and SNU719 were evaluated by Fujisawwa et al. [16]. They found that MC180295 inhibited cell growth and induced apoptosis of those cell lines. Gene expression analysis showed that MC180295 inhibited the growth of EBVaGC cells by suppressing the expression of genes related to DNA repair and the cell cycle.

Yamamoto et al. found that Cys-488 of the SARS-CoV-2 spike protein played a critical role in its Golgi localization and the cleavage of the S1/S2 processing site, which is the target of the cellular protease furin [18,19]. The spike protein of SARS-CoV-2 binds to cellular angiotensin-converting enzyme-2 (ACE2) as the first step of viral cell entry [20]. The spike protein on the ACE2-expressing cell surface induces cell–cell membrane fusion, forming syncytia [21]. The spike protein is cleaved at a specific site (the S1/S2 processing site) by cellular proteases, such as furin, and subsequently exerts its fusogenic activity. Yamamoto et al. reported that Cys488 mutated spike protein failed to localize to the Golgi apparatus and induce syncytia formation. Moreover, this mutant spike was not cleavaged at the S1/S2 processing site [18]. The Cys-488 residue of the spike protein is therefore essential for functional spike protein processing.

Viruses have been reported to reprogram the metabolic profiles of host cells to facilitate self-replication. Herpes simplex virus-1 (HSV-1) causes cold sores, keratitis, meningitis, and encephalitis [22]. To clarify the metabolic interactions between host cells and HSV-1, Huang et al. used liquid chromatography with tandem mass spectrometry (LC–MS/MS) to analyze the metabolic profiles of the human lung fibroblasts cell line (KMB17) infected with HSV-1. They reported that the tryptophan metabolite kynurenine enhanced HSV-1 replication, while 25-hydroxycholesterol and ChOKα (a choline metabolic rate-limiting enzyme) suppressed viral replication [23]. These findings suggest that HSV-1 induces the metabolic reprogramming of host cells to promote or repress viral replication.

HBV is a hepato-tropic enveloped virus with a DNA genome; it is transmitted via the blood and can be sexually acquired [24]. Some HBV-infected individuals develop liver cirrhosis or liver cancer following chronic hepatitis [25]. The addition of N6-methyladenosine (m6A) to mRNA is a post-transcriptional RNA modification related to transcript transport, translation, degradation, and splicing [26]. Murata et al. discovered that HBV RNA is modified by m6A, predominantly in the coding region of HBx. They reported that the mutation of methylation sites or the inhibition of m6A decreased the expression levels of HBV mRNA and HBV surface (HBs) protein [27]. These findings suggest that the cellular m6A modification of HBV RNA is needed for the efficient replication of HBV.

Feline calicivirus (FCV), a single-stranded positive-sense RNA virus, causes immune depression, respiratory diseases, and oral diseases in cats [28]. However, the pathogenic mechanism of FCV remains unclear. Mao et al. determined that FCV infection induced autophagy via the expression of FCV non-structural proteins P30, P32, and P39 [29]. They also observed that altering autophagy influenced the retinoic acid-inducible gene-I (RIG-I) signaling pathway and FCV replication. Their data suggest that FCV facilitates replication by inhibiting the RIG-I pathway through autophagy.

Adeno-associated viruses (AAVs) are small, non-enveloped, single-stranded DNA viruses that infect humans and other primate species. Recombinant AAV (rAAV) has been suggested as a promising candidate as a gene delivery vector for in vivo gene therapy [30]. However, host immune responses to the vector have meant that some hurdles remain for the successful implementation of rAAV-based gene transfer [31,32]. Using transcriptomic analysis, Masri et al. analyzed the activation of inflammatory and antiviral pathways following the rAAV8 infection of monocyte-derived dendritic cells (moDCs) obtained from 12 healthy humans. They reported that rAAV8 was efficiently internalized into human moDCs without significant transcriptomic changes in the moDCs [33]. This finding suggests that rAAV8 does not elicit any inflammatory or antiviral responses in human moDCs.

The Flaviviridae family includes many arthropod-borne viruses, such as dengue virus, West Nile virus, Zika virus, and Japanese encephalitis virus; these often cause life-threatening diseases in humans, with symptoms such as hemorrhaging and encephalitis [34]. The replication of flaviviruses is restricted by IFN-stimulated genes (ISGs) induced by the IFN response. SHFL/C19orf66 is a newly identified ISG that inhibits flavivirus replication [35]. Suzuki et al. summarized the current knowledge relating to the anti-flavivirus activities of SHFL/C19orf66 and discussed the inhibitory molecular mechanism using a predicted SHFL structure generated by AlphaFold2 [36].

The tripartite motif proteins (TRIMs) belong to the E3 ubiquitin ligase family, and each TRIM contains the Really Interesting New Gene (RING) domain and/or B-Box domain [37]. The RING and B-Box domains are necessary for the binding to E2 enzyme and the oligomerization, respectively. TRIM5α is known to be a host anti-retroviral restriction factor that destroys HIV through the interaction with HIV capsid protein [38]. TRIM5α also induces the autophagic degradation of the target proteins. Lin et al. showed that TRIM5α interacted with EBV capsid protein BORF1, and TRIM5α induced BORF1 ubiquitination [39]. Results suggest that TRIM5α destabilizes BORF1 via autophagic pathway.

TRIM21 modulates antiviral immune responses via an interaction involving the ubiquitination of cellular (or viral) proteins. TRIM21 functions as an E3 ubiquitin ligase and acts as a cytosol high-affinity antibody Fc receptor. Li et al. reviewed the role and the importance of TRIM21 in virus infection [40].

Transforming growth factor-β1 (TGF-β1) plays a role in cell proliferation and cell differentiation during the development of the human body. During pregnancy, TGF-β1 plays essential roles not only in cell growth/differentiation but also trophoblast cell invasion and the maintenance of fetal–maternal immune tolerance [41]. The review by Trinh et al. summarizes recent advances in our understanding of the role TGF-β1 plays in viral infection (e.g., Zika virus, rubella virus, human cytomegalovirus, HIV, HBV, HSV, influenza A virus, SARS-CoV-2) during pregnancy, especially in the first trimester [42]. They also discussed possible functions of the Smad pathway in Zika virus infection. 

The review by Itabashi et al. [43] summarized the current knowledge of HTLV-1 mother-to-child transmission (MTCT). HTLV-1 causes adult T-cell leukemia (ATL) and HTLV-1-associated myelopathy/tropical spastic paraparesis [44]. Many ATL patients are infected with HTLV-1 by MTCT through the mother’s milk [45,46]. To prevent HTLV-1 MTCT, authors discussed the nutritional regimens such as exclusive formula feeding and banked human milk. Authors also proposed the necessity for clinical applications of antiretroviral drugs and immunotherapy with vaccines.

In conclusion, the twelve research and four review articles in this Special Issue help to understand the mechanisms underlying the establishment of viral infection, viral replication, and host immune responses through the interactions of cellular and viral molecules. We hope that through an increased understanding of the virus–cell interactions provided, these articles will contribute to the development of new approaches to control and treat viral infections.
